# Quantifying the circulation induced by convective clouds in kilometer‐scale simulations

**DOI:** 10.1002/qj.3992

**Published:** 2021-02-22

**Authors:** Annika Oertel, Sebastian Schemm

**Affiliations:** ^1^ Institute for Atmospheric and Climate Science ETH Zürich Zürich Switzerland; ^2^ Present address: A. Oertel, Institute for Meteorology and Climate Research Karlsruhe Institute of Technology (KIT) Karlsruhe Germany

**Keywords:** cloud‐circulation interaction, diabatic–adiabatic coupling, far‐ and near‐field cloud‐induced circulation, high‐resolution modeling

## Abstract

The complex coupling between the large‐scale atmospheric circulation, which is explicitly resolved in modern numerical weather and climate models, and cloud‐related diabatic processes, which are parameterized, is an important source of error in weather predictions and climate projections. To quantify the interactions between clouds and the large‐scale circulation, a method is employed that attributes a far‐ and near‐field circulation to the cloud system. The method reconstructs the cloud‐induced flow based on estimates of vorticity and divergence over a limited domain and does not require the definition of a background flow. It is subsequently applied to 12‐ and 2‐km simulations of convective clouds, which form within the large‐scale cloud band ahead of the upper‐level jet associated with an extratropical cyclone over the North Atlantic. The cloud‐induced circulation is directed against the jet, reaches up to 10 m·s^−1^, and compares well between both simulations. The flow direction is in agreement with what can be expected from a vorticity dipole that forms in the vicinity of the clouds. Hence, in the presence of embedded convection, the wind speed does not steadily decrease away from the jet, as it does in cloud‐free regions, but exhibits a pronounced negative anomaly, which can now be explained by the cloud‐induced circulation. Furthermore, the direction of the reconstructed circulation suggests that the cloud induces a flow that counteracts its advection by the jet. Convective clouds therefore propagate more slowly than their surroundings, which may affect the distribution of precipitation. The method could be used to compare cloud‐induced flow at different resolutions and between different parameterizations.

## Graphical abstract

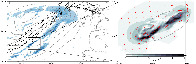

Clouds are not passively moved in the atmosphere by wind. Instead, clouds actively influence the flow field in their immediate vicinity. In this study, the flow field generated by clouds is quantified. It is shown that clouds create a flow that is directed against the direction of the wind in which they grow. Hence, clouds propagate more slowly than a passive object, and they are more stationary. This can influence the regional cloud and precipitation distribution.

## 1 Introduction

Interactions between clouds and the large‐scale atmospheric flow represent one of the main sources of the high level of uncertainty in future projections of regional precipitation changes (Shepherd, 2014; Bony *et al*., 2015). The coupling between adiabatic and diabatic processes in the atmosphere is highly nonlinear and challenges even the latest generation of weather and climate models (Naud *et al*., 2019). For example, convection and cloud‐related diabatic processes, such as condensation and freezing but also turbulence and radiation, significantly alter the temporal evolution of the upper‐level circulation (Saffin *et al*., 2017; Spreitzer *et al*., 2019) and thus provide the foundation for forecast errors to grow (Davies and Didone, 2013; Selz and Craig, 2015; Baumgart *et al*., 2018). The error problem is often rooted in the need to parameterize subgrid scale processes, such as convection, and understand how these processes couple with the resolved‐scale circulation (Stevens and Bony, 2013; Daleu *et al*., 2015; 2016). Thus, it is of interest to diagnose the wind field induced by clouds, in particular their influence on far‐field circulation, in an effort to compare cloud‐circulation interactions across different model resolutions or between different weather and climate models and sets of parameterizations.

The aim of the method adopted here is to assign a fraction of the flow field to the vorticity and divergence that predominate in a limited region. This limited region encompasses, for example, convective clouds, and the vorticity and divergence in the limited region is assumed to result predominantly from the convection and other cloud‐related diabatic physics inside the limited region that drive circulation changes via eddy mass, momentum, and heat fluxes. Especially in a vertically sheared environment, convective momentum transport in shallow and deep convective clouds can play an important role in the modification of the large‐scale flow field (e.g., Tung and Yanai, 2002; Badlan *et al*., 2017; Saggiorato *et al*., 2020). Reconstructing the associated circulation changes from the vorticity and divergence inside the limited region allows for a quantification of the cloud‐induced far‐ and near‐field circulation changes. The reconstruction of the circulation from vorticity and divergence has previously been used in large‐scale atmospheric dynamics (Lynch, 1988; 1989) and is here applied for the first time in a highly unbalanced and turbulent flow situation modeled at kilometer‐scale resolution. The reconstruction method is ideally suited to quantify the cloud‐circulation coupling because it does not rely on the definition of an a priori defined time‐mean or lowpass‐filtered background flow field. The attribution of a flow field to a cloud and related diabatic processes is so far often qualitatively conducted by, for example, subtraction of a background flow from the instantaneous flow (Davies and Didone, 2013) or is accomplished via potential vorticity (PV) inversion assuming balanced flow conditions (Davis and Emanuel, 1991; Davis, 1992). In contrast, the attribution method outlined below allows reconstruction of the flow field without the need to assume balanced background flow conditions. It is independent of the background flow.

The purpose of this article is to present and apply the reconstruction method to a 12‐km and a 2‐km simulation of an extratropical cyclone over the eastern North Atlantic and to quantify the circulation field associated with convective clouds located ahead of the surface cold front embedded in a larger‐scale cloud band. Based on the obtained circulation field, we discuss implications for the precipitation distribution at the surface and the cloud propagation. The 12‐km and the 2‐km simulations are both run without parameterized deep convection, and their associated circulation fields are further compared against a 2‐km simulation that is coarse‐grained to a 12‐km grid spacing. These examples illuminate the insights that can be gained from the reconstruction method and serve as a steppingstone for future research. The reconstruction method is presented in Section 2. In Section 3, the synoptic situation and the model setup are discussed. The presence of circulation anomalies in the vicinity of the jet stream that are associated with convective cloud are discussed in Section 4.1 and 4.2. The associated anomalous mass transport is studied using parcel trajectories in Section 4.3. The cloud‐induced circulation obtained by the reconstruction method is then presented in Section 4.4. We conclude our study in Section 5 and also discuss potential caveats of the outlined approach.

## 2 Reconstructing the cloud‐induced horizontal flow field

Methods for reconstructing the wind field from vorticity and divergence over finite domains have already been discussed by Lynch, (1988; 1989) and Chen and Kuo (1992a; 1992b), and the method adopted here follows the recommendations by Bishop (1996a). We start with the conventional relationships between the horizontal component of vorticity ζ, the streamfunction ψ, the divergence δ, the velocity potential χ, and the horizontal wind **v**,

(1) $$\begin{array}{ll} k\cdot \nabla \times v & =\nabla_{h}^{2}\psi =\zeta \end{array}$$

(2) $$\begin{array}{ll} \nabla_{h}\cdot v & =\nabla_{h}^{2}\chi =\delta \;. \end{array}$$

Subsequent multiplication of Equation 1 by **k** and ∇× and Equation 2 by ∇_*h*_· yields the following expression for the rotational ${\text{v}}_{\psi }$ 1and divergent ${\text{v}}_{\chi }$ parts of the wind,

(3) $$\begin{array}{ll} v_{\psi } & =-\nabla \times \left(\psi k\right) \end{array}$$

(4) $$\begin{array}{ll} v_{\chi } & =\nabla_{h}\chi \;. \end{array}$$

The decomposition of the horizontal wind **v** into rotational and divergent components is exact up to an arbitrary constant known as the harmonic wind (**v**_*ϑ*_),

(5) $$v_{\vartheta }=v-\left(v_{\psi }+v_{\chi }\right),$$

which is both irrotational and divergence‐free. The harmonic wind can be represented by either a streamfunction or a velocity potential. On a sphere or a domain with periodic boundary conditions, the maximum principle for harmonic functions requires that the associated streamfunction or velocity potential of the harmonic wind is constant, and thus **v**_*ϑ*_ = 0 everywhere. For a limited domain, the harmonic wind is irrotational and divergence‐free inside the domain (it acquires maximum and minimum values at the boundaries) and is thus unrelated to the vorticity and divergence sources inside the domain, but becomes part of the flow field that is due to the vorticity and divergence outside of the domain.

Solutions to the Poisson equations Equations (1) and (2) over a limited domain Ω, encompassing, for example, a well‐marked divergence and vorticity anomaly associated with a convective cloud, are given by

(6) $$\begin{array}{ll} \psi_{\Omega }(x,y) & =\frac{1}{2\pi }\int_{\Omega }\zeta (x^{\prime },y^{\prime })\ln(r)dx^{\prime }dy^{\prime } \end{array}$$

(7) $$\begin{array}{ll} \chi_{\Omega }(x,y) & =\frac{1}{2\pi }\int_{\Omega }\delta (x^{\prime },y^{\prime })\ln(r)dx^{\prime }dy^{\prime }\; \end{array}$$

where $\frac{1}{2\pi }\ln(r)$ is the two‐dimensional free‐space Green's function and $r=\sqrt{{\left(x-x^{\prime }\right)}^{2}+{\left(y-y^{\prime }\right)}^{2}}$. In three dimensions, the corresponding Green's function would be $-\frac{1}{4\pi }\frac{1}{r}$. The Green's function accounts for the part of the streamfunction or velocity potential at point **x** = (*x*, *y*) that is due to the vorticity or divergence at point **x***′* = (*x′*, *y′*). The solution of the Poisson equations with the free‐space Green's function thus reconstructs the streamfunction and velocity potential response at point **x** due to the vorticity and the divergence in a finite domain bounded by Ω. The corresponding rotational wind is (Equation 3)

(8) $$\begin{array}{ll} u_{\psi_{\Omega }}(x,y) & =-\frac{\partial \psi_{\Omega }}{\partial y}=\frac{1}{2\pi }\int_{\Omega }\zeta (x^{\prime },y^{\prime })\frac{-(y-y^{\prime })}{r^{2}}dx^{\prime }dy^{\prime } \end{array}$$

(9) $$\begin{array}{ll} v_{\psi_{\Omega }}(x,y) & =\frac{\partial \psi_{\Omega }}{\partial x}=\frac{1}{2\pi }\int_{\Omega }\zeta (x^{\prime },y^{\prime })\frac{\left(x-x^{\prime }\right)}{r^{2}}dx^{\prime }dy^{\prime }\; \end{array}$$

and the divergent wind (Equaion 4) is

(10) $$\begin{array}{ll} u_{\chi_{\Omega }}(x,y) & =\frac{\partial \chi_{\Omega }}{\partial x}=\frac{1}{2\pi }\int_{\Omega }\delta (x^{\prime },y^{\prime })\frac{\left(x-x^{\prime }\right)}{r^{2}}dx^{\prime }dy^{\prime } \end{array}$$

(11) $$\begin{array}{ll} v_{\chi_{\Omega }}(x,y) & =\frac{\partial \chi_{\Omega }}{\partial y}=\frac{1}{2\pi }\int_{\Omega }\delta (x^{\prime },y^{\prime })\frac{\left(y-y^{\prime }\right)}{r^{2}}dx^{\prime }dy^{\prime }\; \end{array}$$

The subscript Ω indicates that these are the wind fields associated with the vorticity and divergence over a limited domain. The sum of both wind components subtracted from the full wind yields the part that is due to the vorticity and divergence outside of the limited domain, which also includes the harmonic part of the flow. We refer to this part of the wind as the environmental wind **v**_**e**_,

(12) $$v_{e}=v-\left(v_{\psi_{\Omega }}+v_{\chi_{\Omega }}\right)\;.$$

For gridded data, the integrals are replaced by the corresponding sum over all grid points, and we estimate the vorticity and divergence using finite differences. Further, as recommend by (Bishop, 1996a), the spherical grid of the employed limited‐area model is mapped onto a flat Cartesian grid using a polar stereographic projection.

## 3 Simulation setup and Lagrangian diagnostics

We apply the attribution method to vorticity and divergence anomalies in a high‐resolution model simulation with a horizontal grid spacing of 0.02° and a medium‐resolution simulation with a horizontal grid spacing of 0.12°, corresponding to approximately 2 and 12 km, respectively. The simulations are performed using the Consortium for Small‐scale Modeling (COSMO) nonhydrostatic limited‐area model (Baldauf *et al*., 2011; Doms and Baldauf, 2018) without parameterized deep convection. The COSMO model is the operational numerical weather prediction model of different European weather services: for example, the Swiss (MeteoSwiss) and the German (DWD) national weather services. It is maintained and developed by the COSMO and documented in detail at www.cosmo‐model.org. Both simulations use the same set of physical parameterizations as the operational MeteoSwiss configuration. Cloud microphysics are parameterized with a one‐moment, six‐category scheme for water vapor (*q*_*v*_), cloud liquid, ice, rain, snow, and graupel content (Baldauf *et al*., 2011). The turbulence parameterization is the level 2.5 scheme of Mellor and Yamada (Mellor and Yamada, 1982). Deep convection is not parameterized, but shallow convection is parameterized with a reduced Tiedke scheme (Tiedtke, 1989). The time‐integration scheme is a two‐time‐level Runge‐Kutta scheme with time‐split treatment of acoustic and gravity waves.

The simulation domain is centered in the eastern North Atlantic and extends from 50°W to 20°E and 30° to 70°N. The simulations are initialized at 0000 UTC September 22, 2016, and the simulated period is 4.5 days, which covers the intense observation periods (IOP) 2 and 3 of the North Atlantic Waveguide and Downstream Impact Experiment (NAWDEX) (Schäfler *et al*., 2018), which could be used to verify the simulation wind anomalies. Observational data collected during the NAWDEX campaign are archived at the German Aerospace Center (DLR) at www.pa.op.dlr.de/nawdex. Initial and lateral boundary conditions of our simulations are taken from the operational European Center for Medium‐Range Weather Forecast (ECMWF) high‐resolution analyses with a horizontal grid spacing of 0.1°. The boundary conditions are updated every 6 hr.

To quantify anomalous air mass transport in the vicinity of the convective clouds, as an additional diagnostic to illustrate the sustained and longer‐lasting effect on the circulation field, we compute forward trajectories with the Lagrangian analysis tool LAGRANTO (Wernli and Davies, 1997; Sprenger and Wernli, 2015). The trajectories are started in the upper troposphere between 6 and 10 km height in a region that encompasses the larger surroundings of the convective cloud (Figure 5a,b) and are calculated from the 3D wind fields of the (a) 12‐km, and (b) 2‐km simulations, which are available with a temporal resolution of 15 min. Subsequently, we approximate the air mass transport by the distance that is covered by the individual trajectories within 3 hr.

## 4 Results

### 4.1 Synoptic overview

**FIGURE 1:**
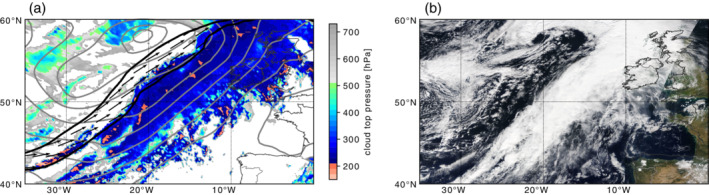
(a) Satellite‐derived cloud top pressure (shading; in hPa) from Meteosat Second Generation Satellites (EUMETSAT; Schmetz *et al.*, 2002), sea‐level pressure (gray contours; steps of 5 hPa), 320 K wind speed (black contour; 50 m·s^−1^), and wind vectors (>50 m·s^−1^) from ERA5 reanalysis (Hersbach *et al.*, 2020) at 1200 UTC September 23, 2016, and (b) satellite image from MODIS overpass at 1225 UTC September 23, 2016 (Data source: NASA Worldview) [Colour figure can be viewed at wileyonlinelibrary.com]

**FIGURE 2:**
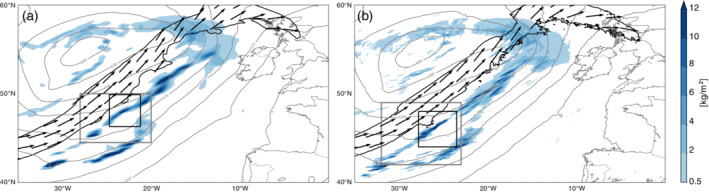
Sea‐level pressure (gray contours; steps of 5 hPa), hourly accumulated precipitation (shading; kg·m^−2^ ·h^−1^), and 320 K wind speed (black contour; 50 m·s^−1^) and wind vectors (>50 m·s^−1^) at (a) 0900 UTC September 23, 2016 for the 12‐km simulation and at (b) 0800 UTC September 23, 2016 for the 2‐km simulation. Additionally shown are two target regions (gray and black boxes) used for the detailed diagnostics in the following sections [Correction added 3 March 2021, after first online publication: The original published version of the article contained an incorrect version of Figure 2. The article has been updated to correct this mistake.] [Colour figure can be viewed at wileyonlinelibrary.com]

The synoptic situation over the Northeastern Atlantic between September 22 and 24, 2016 was characterized by the propagation and development of two mature extratropical cyclones off the British Isles and near Iceland. The southern cyclone, “Vladiana”, had a core pressure of approximately 980 hPa and was attended by an elongated cold front with an extended cloud band covering the cyclone's warm sector (Figure 1). The hourly precipitation sum indicates that the cold front was preceded by two bands of precipitation (dark‐blue shading in Figure 2a,b). The cold‐frontal precipitation formed in the cyclone's warm conveyor belt (WCB), a warm and moist ascending air stream, which ascends ahead of the cold front towards upper levels (Browning, 1986; Wernli and Davies, 1997) and forms the large‐scale and vertically‐extended cloud band with high cloud tops in the cyclone's warm sector (Figure 1b). The upper‐level outflow region of this WCB was successfully probed during NAWDEX by the high‐altitude and long‐range research aircraft (HALO) (Oertel *et al*., 2019).

**FIGURE 3:**
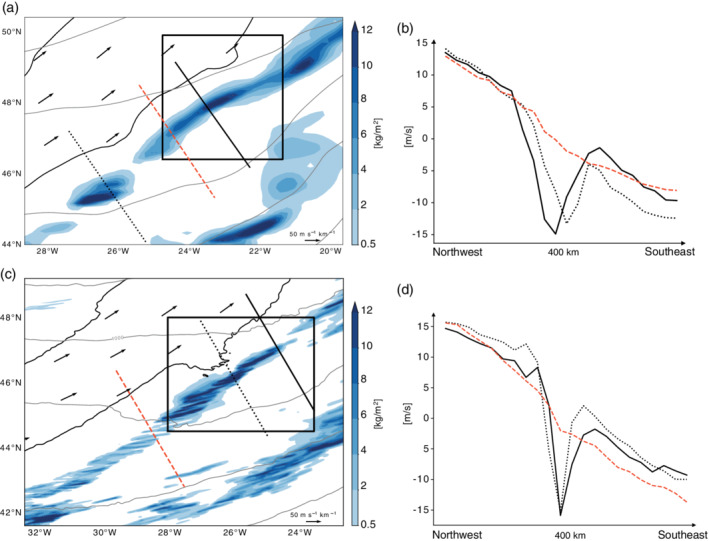
(Left) A zoom into the larger of the two target regions shown in Figure 2 for (a) the 12‐km simulation and (c) the 2‐km simulation. (Right) Wind speed anomaly at the 320 K isentropic level along the three northwest‐to‐southeast oriented sections shown in the left panel for (b) the 12‐km simulation and (d) the 2‐km simulation. The wind speed anomaly is computed relative to wind averaged over the presented domain (m·s^−1^) [Colour figure can be viewed at wileyonlinelibrary.com]

At upper levels above the surface cold front, an elongated SW–NE oriented band of high wind speed indicates the position of the jet axis (black contour in Figures 1a and  2). The wind speed decreases away from the jet axis and precipitation forms ahead of it, where the air is lifted above the surface cold front. A closer look at the frontal precipitation pattern reveals the existence of convective precipitation (Figure 3) embedded in the broader cloud band ahead of the upper‐level jet (Figure 1a,b) – see also Oertel *et al*. (2020). This region is generally known to be populated by convective clouds (Jeyaratnam *et al*. 2020), which are also visible in low cloud‐top temperatures (orange shading in Figure 1a). It is this region ahead of the jet axis where we intend to quantify the interactions between convective clouds and the large‐scale horizontal flow. The exact target regions on which we focus are shown as gray and black boxes in Figure 2. In the next section, the circulation inside these target regions is investigated in greater detail.

### 4.2 The circulation in the vicinity of convective clouds

**FIGURE 4:**
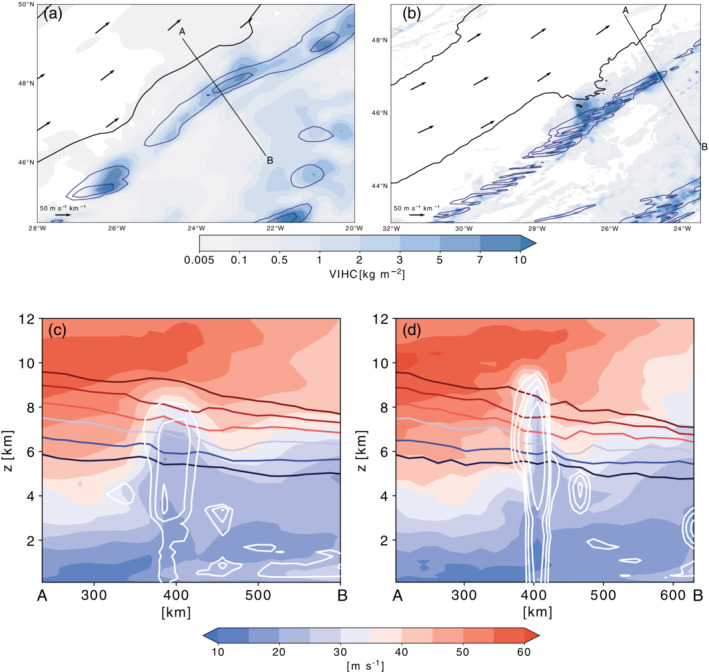
(Top) Hourly accumulated precipitation (contours; at 3 and 10 kg·m^−2^ ·hr^−1^) and vertically integrated hydrometeor content (blue shading; kg·m^−2^) for (a) the 12‐km simulation and (b) the 2‐km simulation. (Bottom) Vertical cross‐sections of wind speed (shading), total hydrometeor content (white contours at 0.3, 0.6, 1.0, and 2.0 g·kg^−1^), and isentropes (at 315–325 K in steps of 2 K) along the northwest‐to‐southeast oriented cross‐section shown in the upper panel (a,b) [Correction added 3 March 2021, after first online publication: The original published version of the article contained incorrect versions of Figures 4 and 5. The article has been updated to correct this mistake.] [Colour figure can be viewed at wileyonlinelibrary.com]

Firstly, we discuss the circulation and related flow anomalies in the vicinity of convective clouds qualitatively. The upper‐level wind speed decreases by definition away from the jet axis. In regions where surface precipitation is low or absent, the wind speed decreases almost linearly away from the jet axis (for example, along the red sections in Figure 3). However, in regions where surface precipitation is high, the steady decrease of the wind speed away from the jet axis is locally interrupted (for example, along the two black sections shown in Figure 3a,c) before the wind speed increases again at distances farther away from the jet axis (Figure 3b,d). These localized negative anomalies in the wind speed, which are found in both the 2‐ and 12‐km simulations, coincide not only with enhanced values of surface precipitation (blue shading in Figure 3a,b) but also with the presence of convective clouds, which are indicated by high values of vertically integrated hydrometeor content (blue shading in Figure 4a,b). A vertical cross‐section highlights the existence of convective clouds and the regions of reduced wind speed (Figure 4c,d) ahead of the upper‐level jet stream (red shading in Figure 4c,d). The convective cloud and the local wind speed anomaly coincide, and extend vertically from approximately 3–10 km, with the wind speed reduction being particularly large in the upper troposphere near the 320 K isentropic surface (Figure 4c,d). The existence of these small‐scale wind anomalies in the vicinity of convective clouds is not overly surprising, given that convective clouds are sources and sinks of vorticity and divergence, due to the upright mass transport and the associated column stretching, and tilting of vorticity. The strong ascent in convective clouds results in a low‐level convergence and upper‐level divergence pattern due to mass conservation. The non‐divergent flow is characterized by a distinct mesoscale quasi‐horizontal vertical vorticity dipole centered around the convective updrafts, which is also seen in our simulations (see below the detailed discussion in Section 4.4.). The formation of mesoscale vorticity dipoles near clouds has been investigated thoroughly from the PV perspective (e.g., Chagnon and Gray, 2009; Schemm, 2013; Oertel *et al*., 2020; 2021; Müller *et al*., 2020), but it can also be understood from the vorticity perspective. The vorticity equation is given by (Holton, 2004, p. 101),

(13) $$\begin{array}{llll} \frac{D}{Dt}\left(\zeta +f\right) & =-\left(\zeta +f\right)\left(\frac{\partial u}{\partial x}+\frac{\partial v}{\partial y}\right)-\left(\frac{\partial w}{\partial x}\frac{\partial v}{\partial z}-\frac{\partial w}{\partial y}\frac{\partial u}{\partial z}\right)\; & & \\ & \;+\frac{1}{\rho^{2}}\left(\frac{\partial \rho }{\partial x}\frac{\partial p}{\partial y}-\frac{\partial \rho }{\partial y}\frac{\partial p}{\partial x}\right)\;. \end{array}$$

**FIGURE 5:**
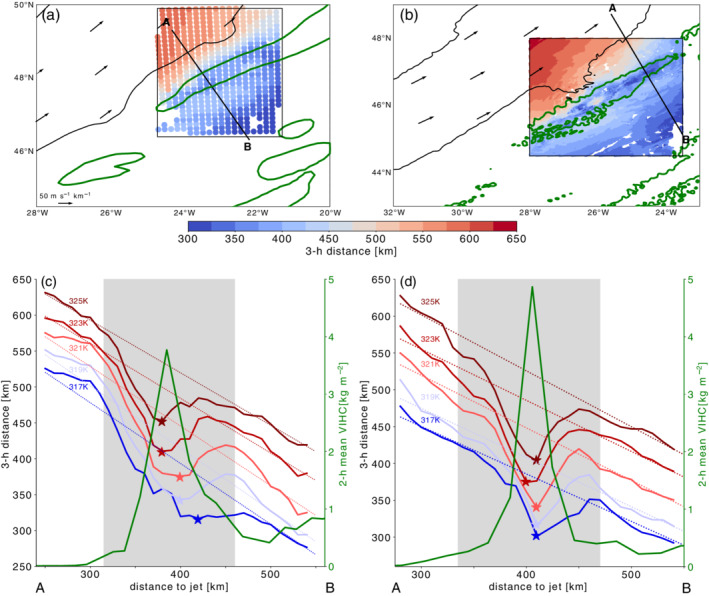
(Top) Distance covered in 3 hrs (shading; km) by parcel trajectories started on the 320 K isentropic level inside the target region (black box in Figure 1) for the (a) 12‐km simulation and (b) the 2‐km simulation; the core of the convective cloud is indicated by the 2 kg·m^−2^ contour of the 2‐hr mean of the vertically integrated hydrometeor content (green). (Bottom) The 3‐hr covered distance as function of the distance to the jet for the (c) 12‐km simulation and (d) the 2‐km simulation; Trajectories are released at different levels (317–325 K in steps of 2 K) along the black NW–SE oriented section shown in (a,b); 2‐hr mean of the vertically integrated hydrometeor content (green line; in kg·m^−2^); colored asterisks indicate the local minimum of the covered distance inside the cloud region (gray shading). Further shown is the result of a linear fit to the 3‐hr distance as function of the distance to the jet (dashed lines) excluding parcel trajectories released inside the cloud region [Colour figure can be viewed at wileyonlinelibrary.com]

The forcing terms on the right are the contributions from divergence, tilting, and from the solenoid. Vorticity tilting is assumed to cause the formation of mesoscale vorticity dipoles in the vicinity of clouds. In the presence of a maximum in convection, which denotes a local maximum in *w*, that is located within a vertically sheared jet environment (*∂v*/*∂z* > 0, *∂u*/*∂z* > 0), it is the change of the sign of the horizontal gradient of *w* (i.e., *∂w*/*∂x* and *∂w*/*∂y* ) that leads to the formation of opposite vorticity tendencies to either side of the updraft maximum. As a result of the convective updraft, horizontal vorticity is tilted into vertical vorticity (see also the schematic in Figure 4.12 in Holton (2004), p. 102). In the following, we first explore the flow distortion caused by the convective clouds using air parcel trajectories before we quantify the cloud‐induced circulation associated with the vorticity and divergence anomalies with the reconstruction method described earlier.

### 4.3 Lagrangian transport of air in the vicinity of convective clouds

In this section, we analyze the circulation in the vicinity of convective clouds more deeply using Lagrangian air parcel trajectories. The convective clouds, which form ahead of the upper‐level jet, persist for several hours. Figure 5 shows the distance covered in 3 hr by parcel trajectories released at every grid point on the 320 K level in the target region shown in Figure 2. The covered distance is projected onto the starting location (color shading in Figure 5a,b). The general pattern of the covered distance agrees with what would be expected from the wind field: it decreases almost linearly with increasing distance away from the jet axis, but the steady decrease is interrupted by local minima (darker‐blue shading in Figure 3a,b) in the area of the convective cloud (green contour in Figure 3a,b). The covered distance of parcel trajectories released along the cross‐section shown in Figure 4a,b as a function of the distance to the jet axis is shown for different isentropic levels in Figure 5c,d. The covered distance decreases away from the jet axis, but the nearly linear decrease is interrupted by the convective cloud, which is located 300–450 km away from the jet axis (green line Figure 5c,d). In the realm of the convective cloud (gray shading in Figure 5c,d), the distance covered by the trajectories rapidly decreases before it increases again. In the 2‐km simulation, the air parcels at, for example, the 325 K level (solid dark‐red line in Figure 5d), which are located 400 km away from the jet axis, cover only 400 km in 3 hrs. This is approximately 150 km less than predicted by a simple linear fit (dashed dark‐red line in Figure 5d). The linear fit is based on all covered distances for all trajectories released outside of the convective cloud area and provides a rough estimate of the expected covered distance in the absence of the cloud. For example, air parcels at the 318 K level that are located 500 km away from the jet axis cover a distance of approximately 450 km, which is in agreement with the expectation from the linear fit. In contrast, inside the cloud region at a distance to the jet of only 400 km, the air parcels started on the same level cover only 370 km in 3 hrs, which is approximately 100 km less than expected from the linear fit.

The difference between the expectation and the observed distance shows that the observed distance covered by air parcels in the cloud‐affected region is reduced by approximately 70–150 km, which corresponds to approximately 20–40% of the total distance covered in 3 hrs. Thus, the 3‐hr distance in this example is reduced by one‐third because of the presence of the convective cloud. In the next section, we use the attribution method to quantify the circulation influence of the convective cloud.

### 4.4 Reconstructing the cloud‐induced circulation

**FIGURE 6:**
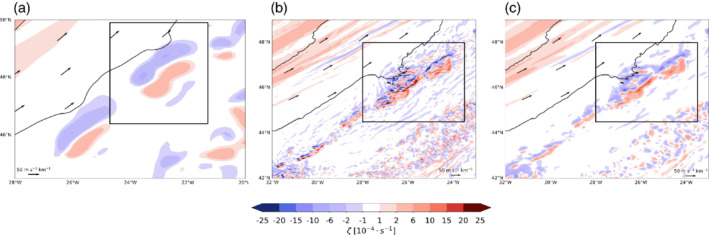
Relative vorticity at the 320 K level (s^−1^) for (a) the 12‐km simulation, (b) the 2‐km simulation, and (c) the 2‐km simulation after coarse‐graining of the wind to 12 km [Colour figure can be viewed at wileyonlinelibrary.com]

In the previous sections, we qualitatively described the circulation in the vicinity of convective clouds (Section 4.2.) and analyzed the related flow anomalies using parcel trajectories (Section 4.3). In this section, we turn our attention to the quantification of the cloud‐induced circulation anomalies. The relative vorticity in the vicinity of the convective region displays a dipole structure (see Section 4.2. and Equation 13), with negative vorticity (blue shading in Figure 6) found on the side of the convection that is towards the jet axis and positive vorticity (red shading in Figure 6) farther away from the jet axis to the east of the convective cloud. Several of these vorticity dipole patterns are located ahead of the jet axis (Figure 6a). The vorticity dipoles are centered around the convective cloud center and tend to align along a common axis, which corresponds to the precipitation band found, for example, in Figure 2 and is parallel to the jet axis. In the 2‐km simulation (Figure 6b), the vorticity displays a stronger fine‐scale structure with stronger vorticity anomalies; however, overall, we find that the vorticity dipole patterns and their alignment are common features of both simulations. The vorticity dipole pattern is also preserved under coarse‐graining from the 2‐km grid to a 12‐km grid (Figure 6c). In fact, the larger‐scale vorticity dipole structure becomes more distinct after the coarse‐graining, and the magnitude of the vorticity anomalies is lower and more similar to the 12‐km simulation. The vorticity patterns help to understand the localized reduction of the wind speed, which were previously identified in this region (Figure 3), as well as the reduced mass transport (Figure 5). The anticyclonic circulation around the negative vorticity maximum and the cyclonic circulation around the positive vorticity maximum will jointly induce a flow, which is directed along their common axis and against the direction of the jet. While it is not overly surprising that such vorticity anomalies exist in the realm of convective activity, it is the goal of the attribution method presented here to quantify the wind field associated with such vorticity dipoles.

**FIGURE 7:**
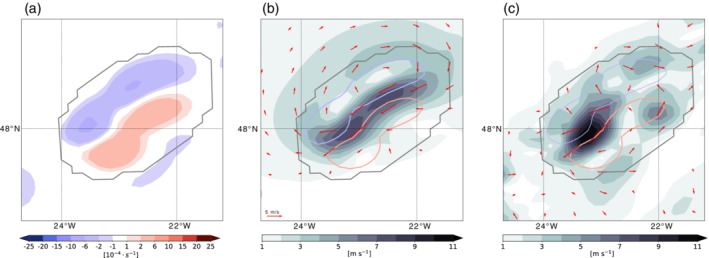
(a) Relative vorticity on the 320 K isentropic level (shading; s^−1^) and the cloud box (gray contour) that is centered on the convective cloud in the 12‐km simulation. (b) Reconstructed rotational wind field (red vectors; m·s^−1^), the corresponding wind magnitude (shading), and two selected negative (blue) and positive (red) vorticity contours (± 2×10^−4^ s^−1^). (c) Wind anomalies taken relative to the 2‐hr centered mean wind [Colour figure can be viewed at wileyonlinelibrary.com]

To apply the attribution method, we first define a limited domain that is chosen to encompass the area of convective activity. We regard the physical processes associated with the convective cloud as the main sources of vorticity and divergence within the finite domain, which we refer to as the cloud box. The cloud box is centered on the convective cloud and encompasses the vorticity dipole pattern (gray ellipse in Figure 7a). The reconstructed rotational wind related to the vorticity source inside the cloud box will be referred to as the cloud‐induced rotational wind.

The strength of the cloud‐induced rotational wind in the 12‐km simulation is strongest between the vorticity dipole (Figure 7b), where the circulation of the positive and negative vorticity add up. The wind speed inside the cloud box, which is directed against the direction of the jet, is on the order of 6–8 m·s^−1^ and thus explains partly the reduced wind speed that coincides with the convective cloud band (Figure 3b). To the northwest outside of the cloud box, the far‐field wind is anticyclonic and up to 2–3 m·s^−1^. To the southeast, the weaker positive vorticity anomaly induces no well‐marked far‐field cyclonic wind field. The wind field that is due to the divergence in the cloud box is several orders of magnitude smaller compared with that related to the vorticity (not shown). The total reconstructed wind is therefore almost identical to the wind shown in Figure 7b.

Figure 7c shows the result of the corresponding wind anomaly obtained after subtracting the centered 2‐hr mean wind. The 2‐hr period was chosen after a series of manual inspections, with the goal of obtaining wind anomalies that match those obtained from the attribution method with respect to magnitude. Longer time periods result in wind anomalies that exhibit large magnitudes due to the propagation of the combined jet‐front system. In contrast to the wind anomalies obtained from the attribution method, the wind anomalies' deviation from the 2‐hr mean provides a rather vague indication of the cloud‐induced circulation (Figure 7c). The wind is again strongest between the two vorticity anomalies, though more confined to the southwestern sector of the target region. Qualitatively, the obtained anomalies indeed also allow inference of a cyclonic and anticyclonic circulation around the cloud box; however, the anomalies are less clear compared with those obtained from the attribution method. Further, the choice of the 2‐hr mean as the most appropriate reference period is based in the first place on the comparison with the reconstructed wind obtained from the attribution method. The strength of the attribution method is therefore that it reveals qualitatively as well as quantitatively how the convective cloud influences the local and far‐field circulation

**FIGURE 8:**
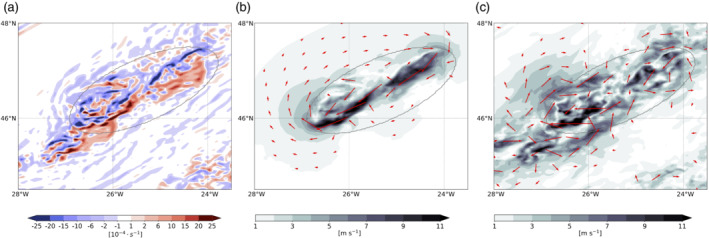
(a) Relative vorticity on the 320 K isentropic level (shading; s^−1^) and the cloud box (gray contour) that is centered on the convective cloud in the 2‐km simulation. (b) Reconstructed rotational wind field (red vectors; m·s^−1^) and the corresponding wind magnitude (shading). (c) Wind anomalies taken relative to the 2‐hr centered mean wind [Colour figure can be viewed at wileyonlinelibrary.com]

For the 2‐km simulation, it is more challenging to isolate a single vorticity dipole (Figure 8) compared with the 12‐km simulation, but the well‐aligned positive and negative vorticity bands still allow for quantification of the cloud‐induced circulation. Similar as for the 12‐km simulation, we define a cloud box that is centered on a region of convective activity (gray ellipse in Figure 8b), for which we previously identified a nonmonotonic decrease in the wind speed away from the jet axis (Figure 3c) and high cloud hydrometeor content (Figure 4b). The circulation attributed to the vorticity source inside the cloud box is in reasonably good agreement with the results obtained from the 12‐km simulation (Figure 8b). Again, the cloud‐induced rotational wind is directed against the direction of the jet and is strongest between the vorticity dipole along the axis of alignment, with wind speed of up to 5–10 m·s^−1^, which is approximately 10–25 % of the wind speed in the jet region. The anticyclonic circulation extends well outside of the cloud box, while the cyclonic circulation is much weaker, as already observed in the 12‐km simulation. This points towards the dominance of the negative vorticity anomalies inside the cloud box. Further interesting details are, for example, the two wind‐speed maxima, the first in the northeastern (upper right) corner of the cloud box and a second in the southwestern (lower left) corner of the cloud box (Figure 8b). This is in very good agreement with the fact that this region indeed encompasses two centers of high vertically integrated hydrometeor content (Figure 4b), and thus also two related vorticity dipoles (Figure 8a). The wind anomaly relative to the 2‐hr mean wind is much more fringed: in particular, the elongated band of larger magnitude wind speed (shading in Figure 8c) is less distinct and not entirely aligned with the precipitation band. The wind anomaly vectors nevertheless offer a vague idea of the induced circulation, but the pattern is much less clear if compared against the reconstructed rotational wind in Figure 8b.

**FIGURE 9:**
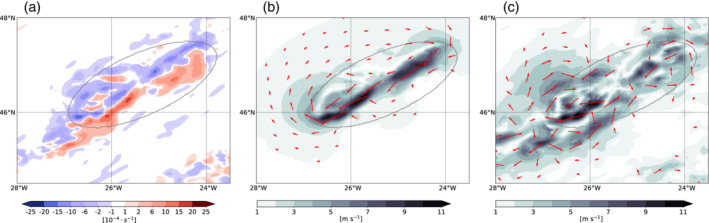
(a) Relative vorticity on the 320 K isentropic level (shading; s^−1^) and the cloud box (gray contour) that is centered on the convective cloud in a 2‐km simulation coarse‐grained to a 12‐km grid spacing. (b) Reconstructed rotational wind field (red vectors; m·s^−1^) and the corresponding wind magnitude (shading). (c) Wind anomalies taken relative to the 2‐hr centered mean wind [Colour figure can be viewed at wileyonlinelibrary.com]

We also applied the attribution method to the wind field from the 2‐km simulation that is coarse‐grained to a 12‐km grid (Figure 9b). The reconstructed wind barely differs between the 2‐km and the coarse‐grained wind fields, which suggests that the integrated effect of the vorticity in the cloud box on the far‐field circulation does not hinge on the fine‐scale vorticity structure. Overall, the local and far‐field circulation influences of the convective cloud are fairly well retained under a coarse‐graining of the data.

**FIGURE 10:**
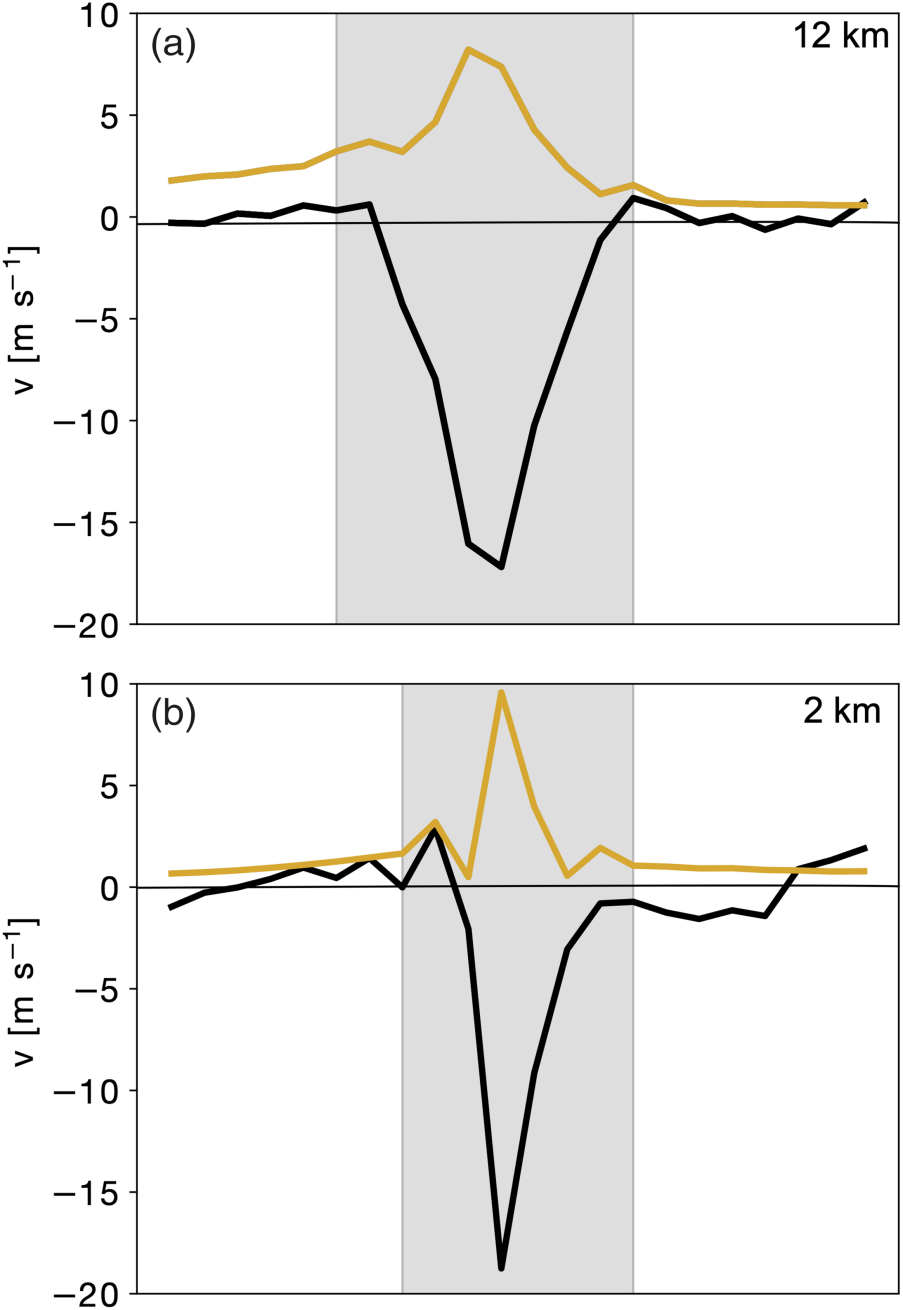
Total reconstructed wind speed (orange line) and wind speed anomaly (black line) at the 320 K isentropic level along the NW–SE oriented section shown in Figure 3 a,b for (a) the 12‐km simulation and (b) the 2‐km simulation. The cloud region is indicated by the gray shading. The wind speed anomaly (black line) is computed as the difference between the full wind speed and a hypothetical linear decrease of the wind speed away from the jet [Colour figure can be viewed at wileyonlinelibrary.com]

Figure 10 shows the total cloud‐induced circulation and the deviation of the full wind speed from a hypothetical linear decrease of the wind speed away from the jet (Figure 3b,d, red line) along the cross‐section shown in Figure 4a,b (black line). In this region, the total reconstructed wind field is on the order of 8 m·s^−1^ (orange line in Figure 10a) in the 12‐km simulation and 10 m·s^−1^ in the 2‐km simulation. Hence, the attribution method explains approximately 50% of the observed wind speed reduction in both simulations, suggesting that the estimated influence of the cloud‐induced circulation might be somewhat underestimated by the method. We discuss limitations of the reconstruction method that could help explain the remaining fraction of the flow anomaly in the summary and discussion.

**FIGURE 11:**
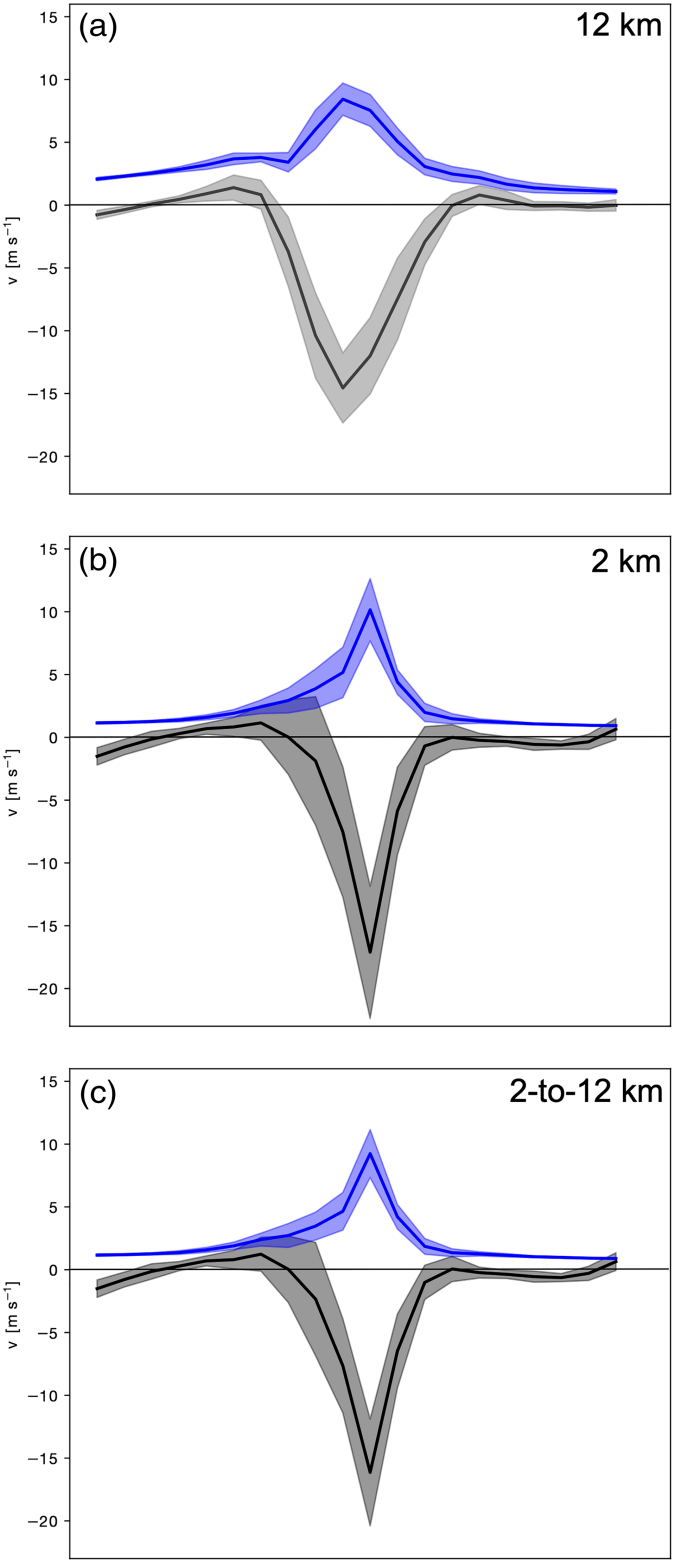
As Figure 10, but averaged over several cross‐sections across the vorticity dipole shown in Figures 7, 8, 9. Shown are the total cloud‐induced wind speed (blue) and the wind speed anomaly relative to a hypothetical linear decrease of the wind speed away from the jet (gray) for the (a) 12‐km simulation, **(**b) 2‐km simulation, and (c) 2‐km simulation after coarse‐graining of the wind field to a 12‐km grid. The mean is shown by a solid line, and the shading represents ±1 standard deviation [Colour figure can be viewed at wileyonlinelibrary.com]

Finally, the total cloud‐induced circulation is evaluated systematically along several NW–SE oriented cross‐sections across the vorticity dipoles shown in Figures 7, 8, 9. Both the wind speed reduction and the reconstructed wind are relatively robust if the result is averaged across multiple cross‐sections (Figure 11). While it is not surprising that the reconstructed wind for the 12‐km simulation, where the vorticity anomaly field is relatively homogeneous (Figure 7a), varies little along several cross‐sections, it is remarkable that the magnitude and standard deviation of the wind speed anomaly and the total reconstructed wind from the 2‐km simulation (Figure 11b) are comparable to those of the 12‐km simulation (Figure 11a), although the variance of the vorticity along the cross‐sections through the cloud region is substantially larger for the 2‐km simulation. In particular, the reconstructed wind field from the 2‐km simulation (Figure 11b) is almost identical to the wind field retained after coarse‐graining to a 12‐km grid (Figure 11c), although the small‐scale vorticity (and divergence) patterns are much more variable on the original 2‐km grid (cf. Figures 8a and 9a). This again emphasizes the integrated effect of vorticity and divergence inside the cloud box on the wind field and suggests that the cloud‐induced circulation is not very sensitive to the detailed small‐scale structures of vorticity and divergence within the selected cloud box.

## 5 Summary and discussion

Because of their complex, highly nonlinear, and chaotic nature, cloud‐circulation interactions challenge even the latest generation of high‐resolution weather and climate models and are, not surprisingly, an important source of error in numerical models of the atmospheric circulation. In this study, we apply a method to reconstruct the wind field associated with convective clouds in an effort to quantify how clouds affect the near‐ and far‐field circulation in their surroundings. The aim of the method, which was previously applied to large‐scale circulating systems (Bishop, 1996b; Chaboureau and Thorpe, 1999), is to assign a fraction of the flow field to the vorticity and divergence associated with a cloud system within a limited domain. Over this limited domain, the wind is reconstructed using the free‐space Green's function of the associated Poisson equations for streamfunction and velocity potential. The method does not require artificial boundary conditions imposed on the limited domain or balanced assumptions of the background flow, as is the case for PV inversion.

We applied the reconstruction method to a number of convective clouds located above a surface cold front and ahead of an upper‐level jet over the North Atlantic in a 12‐km and a 2‐km simulation using the local weather prediction model COSMO (Baldauf *et al*., 2011). Deep convection is not parameterized in either simulation. In the vicinity of convective clouds, which all align along the cold front, we observe the formation of vorticity dipoles. Negative vorticity is located to the west and positive vorticity to the east of the cloud. The combined action of both vorticity anomalies is a flow field that is directed against the direction of the jet stream. Consequently, in the presence of embedded convection, the wind speed does not linearly decrease away from the jet, as it does in cloud‐free conditions, but strongly reduces before it increases again at distances farther away from the jet axis. This narrow and elongated wind speed reduction is not only a transient feature but is maintained for several hours ahead of the jet, and hence reduces the air mass transport in the cloud region. The reconstruction method allows for a quantification of the cloud‐induced circulation and thus the contribution of the clouds to this circulation anomaly. In both simulations, we can attribute a wind speed of up to 10 m·s^−1^ to the convective clouds. The total cloud‐induced circulation is primarily directed against the mean flow, in agreement with the vorticity anomalies in the vicinity of the cloud. It is dominated by the rotational wind that is associated with the vorticity in the cloud region, while the reconstructed irrotational wind due to the divergence is substantially smaller. The cloud‐induced circulation is relatively robust when compared between the different simulations and is also retained when the 2‐km simulation is coarse‐grained to a grid‐spacing of 12 km. The reconstructed circulation suggests that convective clouds induced a flow field that slows down their advection by the mean flow. Hence, the clouds are more stationary compared with their surroundings, which affects the local cloud distribution and could potentially influence the accumulated surface precipitation pattern. It would be intriguing to connect the cloud motion to measured precipitation patterns. Also, we believe it could be insightful to apply the reconstruction method to different cloud types and also at different latitudes, such as tropical shallow clouds (Bony *et al*., 2017), which are known to interact strongly with wind speed (Nuijens and Stevens, 2012), or it could be used to compare the cloud‐induced flow across different models (Maloney *et al*., 2019).

Qualitatively, our results are also in good agreement with what would be expected from “PV thinking” (Hoskins *et al*., 1985), based on which we would regard clouds as PV sources and sinks. This is similar to the interpretation in this study, that clouds are major sources of mesoscale flow divergence and vorticity. The PV invertibility principle would in general also allow for the reconstruction of the wind and even the temperature and pressure field. The vorticity dipoles analyzed here (Figure 7) indeed correspond to dipoles of negative and positive PV (see e.g., Figure 7c in Oertel *et al*. (2020)). The circulation expected from the perspective of PV is thus directed against the jet, because the positive PV anomaly is associated with a cyclonic circulation, and the negative PV anomaly with an anticyclonic circulation. However, for quantification of the associated wind field, the underlying PV inversion requires the formulation of suitable balanced background flow conditions, which in most studies is taken to be Charney's balance equation (Davis, 1992). In a highly unbalanced mesoscale flow situation as the one analyzed here, such an approach is, however, questionable.

Finally, the main limitation of the reconstruction method is its two‐dimensional nature, and thus the assumption that the horizontal wind can be reconstructed from the vertical component of vorticity and horizontal divergence alone. An extension of the method to three dimensions would allow for additional quantification of the influence of the two horizontal vorticity components on the horizontal wind field. Moreover, we recommend testing the added value of high‐order methods to estimates of vorticity and divergence inside the limited domain. In particular, the utilized finite‐difference estimate of divergence results in a highly unstructured field and eventually could be improved using more accurate methods.

## Footnotes

1 With the help of the identity for the Laplacian vector ∇ × (∇ × **v**) = ∇(∇ · **v**) − ∇^2^**v** and the assumption that the first term on the r.h.s is zero by definition of a rotational wind.

## References

[qj3992-bib-0001] Badlan, R.L. , Lane, T.P. , Moncrieff, M.W. and Jakob, C. (2017) Insights into convective momentum transport and its parametrization from idealized simulations of organized convection. Quarterly Journal of the Royal Meteorological Society, 143, 2687–2702.

[qj3992-bib-0002] Baldauf, M. , Seifert, A. , Förstner, J. , Majewski, D. , Raschendorfer, M. and Reinhardt, T. (2011) Operational convective‐scale numerical weather prediction with the COSMO model: description and sensitivities. Monthly Weather Review, 139, 3887–3905.

[qj3992-bib-0003] Baumgart, M. , Riemer, M. , Wirth, V. , Teubler, F. and Lang, S.T.K. (2018) Potential vorticity dynamics of forecast errors: a quantitative case study. Monthly Weather Review, 146, 1405–1425.

[qj3992-bib-0004] Bishop, C.H. (1996a) Domain‐independent attribution. Part I: reconstructing the wind from estimates of vorticity and divergence using free space Green's functions. Journal of the Atmospheric Sciences, 53, 241–252.

[qj3992-bib-0005] Bishop, C.H. (1996b) Domain‐independent attribution. Part II: its value in the verification of dynamical theories of frontal waves and frontogenesis. Journal of the Atmospheric Sciences, 53, 253–262.

[qj3992-bib-0006] Bony, S. , Stevens, B. , Ament, F. , Bigorre, S. , Chazette, P. , Crewell, S. , Delanoë, J. , Emanuel, K. , Farrell, D. , Flamant, C. , Gross, S. , Hirsch, L. , Karstensen, J. , Mayer, B. , Nuijens, L. , Ruppert, J.H. , Sandu, I. , Siebesma, P. , Speich, S. , Szczap, F. , Totems, J. , Vogel, R. , Wendisch, M. and Wirth, M. (2017) EUREC4A: a field campaign to elucidate the couplings between clouds, convection and circulation. Surveys in Geophysics, 38, 1529–1568. 31997845 10.1007/s10712-017-9428-0 PMC6956937

[qj3992-bib-0007] Bony, S. , Stevens, B. , Frierson, D.M. , Jakob, C. , Kageyama, M. , Pincus, R. , Shepherd, T.G. , Sherwood, S.C. , Siebesma, A.P. , Sobel, A.H. , Watanabe, M. and Webb, M.J. (2015) Clouds, circulation and climate sensitivity. Nature Geoscience, 8, 261–268.

[qj3992-bib-0008] Browning, K.A. (1986) Conceptual models of precipitation systems. Weather and Forecasting, 1, 23–41.

[qj3992-bib-0009] Chaboureau, J.‐P. and Thorpe, A.J. (1999) Frontogenesis and the development of secondary wave cyclones in FASTEX. Quarterly Journal of the Royal Meteorological Society, 125, 925–940.

[qj3992-bib-0010] Chagnon, J.M. and Gray, S.L. (2009) Horizontal potential vorticity dipoles on the convective storm scale. Quarterly Journal of the Royal Meteorological Society, 135, 1392–1408.

[qj3992-bib-0011] Chen, Q.‐S. and Kuo, Y.‐H. (1992a) A consistency condition for wind‐field reconstruction in a limited area and a harmonic‐cosine series expansion. Monthly Weather Review, 120, 2653–2670.

[qj3992-bib-0012] Chen, Q.‐S. and Kuo, Y.‐H. (1992b) A harmonic‐sine series expansion and its application to partitioning and reconstruction problems in a limited area. Monthly Weather Review, 120, 91–112.

[qj3992-bib-0013] Daleu, C.L. , Plant, R.S. , Woolnough, S.J. , Sessions, S. , Herman, M.J. , Sobel, A. , Wang, S. , Kim, D. , Cheng, A. , Bellon, G. , Peyrille, P. , Ferry, F. , Siebesma, P. and van Ulft, L. (2015) Intercomparison of methods of coupling between convection and large‐scale circulation: 1. comparison over uniform surface conditions. Journal of Advances in Modeling Earth Systems, 7, 1576–1601. 27642500 10.1002/2015MS000468 PMC5006259

[qj3992-bib-0014] Daleu, C.L. , Plant, R.S. , Woolnough, S.J. , Sessions, S. , Herman, M.J. , Sobel, A. , Wang, S. , Kim, D. , Cheng, A. , Bellon, G. , Peyrille, P. , Ferry, F. , Siebesma, P. and van Ulft, L. (2016) Intercomparison of methods of coupling between convection and large‐scale circulation: 2. comparison over nonuniform surface conditions. Journal of Advances in Modeling Earth Systems, 8, 387–405. 27642501 10.1002/2015MS000570 PMC5008117

[qj3992-bib-0015] Davies, H.C. and Didone, M. (2013) Diagnosis and dynamics of forecast error growth. Monthly Weather Review, 141, 2483–2501.

[qj3992-bib-0016] Davis, C.A. (1992) Piecewise potential vorticity inversion. Journal of the Atmospheric Sciences, 49, 1397–1411.

[qj3992-bib-0017] Davis, C.A. and Emanuel, K.A. (1991) Potential vorticity diagnostics of cyclogenesis. Monthly Weather Review, 119, 1929–1953.

[qj3992-bib-0018] Doms, G. and Baldauf, M. (2018) A Description of the Non‐hydrostatic Regional COSMO Model. Part I: Dynamics and Numerics. Offenbach: Deutscher Wetterdienst. Available at: www2.cosmo‐model.org/content/model/documentation/core/cosmoDyncsNumcs.pdf [Accessed October, 2019]. Report COSMO‐Model 5.5.

[qj3992-bib-0019] Hersbach, H. , Bell, B. , Berrisford, P. , Hirahara, S. , Horányi, A. , Muñoz‐Sabater, J. , Nicolas, J. , Peubey, C. , Radu, R. , Schepers, D. , Simmons, A. , Soci, C. , Abdalla, S. , Abellan, X. , Balsamo, G. , Bechtold, P. , Biavati, G. , Bidlot, J. , Bonavita, M. , De Chiara, G. , Dahlgren, P. , Dee, D. , Diamantakis, M. , Dragani, R. , Flemming, J. , Forbes, R. , Fuentes, M. , Geer, A. , Haimberger, L. , Healy, S. , Hogan, R.J. , Hólm, E. , Janisková, M. , Keeley, S. , Laloyaux, P. , Lopez, P. , Lupu, C. , Radnoti, G. , de Rosnay, P. , Rozum, I. , Vamborg, F. , Villaume, S. and Thépaut, J.‐N. (2020) The ERA5 global reanalysis. Quarterly Journal of the Royal Meteorological Society, 146, 1999–2049.

[qj3992-bib-0020] Holton, J.R. (2004) An Introduction into Dynamic Meteorology Vol. 88, 4th ed., p. 535. London: Elsevier Academic Press.

[qj3992-bib-0021] Hoskins, B.J. , McIntyre, M.E. and Robertson, A.W. (1985) On the use and significance of isentropic potential vorticity maps. Quarterly Journal of the Royal Meteorological Society, 111, 877–946.

[qj3992-bib-0022] Jeyaratnam, J. , Booth, J.F. , Naud, C.M. , Luo, Z.J. and Homeyer, C.R. (2020) Upright convection in extratropical cyclones: a survey using ground‐based radar data over the United States. Geophysical Research Letters, 47, e2019GL086620.

[qj3992-bib-0023] Lynch, P. (1988) Deducing the wind from vorticity and divergence. Monthly Weather Review, 116, 86–93.

[qj3992-bib-0024] Lynch, P. (1989) Partitioning the wind in a limited domain. Monthly Weather Review, 117, 1492–1500.

[qj3992-bib-0025] Maloney, E.D. , Gettelman, A. , Ming, Y. , Neelin, J.D. , Barrie, D. , Mariotti, A. , Chen, C.‐C. , Coleman, D.R.B. , Kuo, Y.‐H. , Singh, B. , Annamalai, H. , Berg, A. , Booth, J.F. , Camargo, S.J. , Dai, A. , Gonzalez, A. , Hafner, J. , Jiang, X. , Jing, X. , Kim, D. , Kumar, A. , Moon, Y. , Naud, C.M. , Sobel, A.H. , Suzuki, K. , Wang, F. , Wang, J. , Wing, A.A. , Xu, X. and Zhao, M. (2019) Process‐oriented evaluation of climate and weather forecasting models. Bulletin of the American Meteorological Society, 100, 1665–1686.

[qj3992-bib-0026] Mellor, G.L. and Yamada, T. (1982) Development of a turbulence closure model for geophysical fluid problems. Reviews of Geophysics, 20, 851–875.

[qj3992-bib-0027] Müller, A. , Niedrich, B. and Névir, P. (2020) Three‐dimensional potential vorticity structures for extreme precipitation events on the convective scale. Tellus A: Dynamic Meteorology and Oceanography, 72, 1–20.

[qj3992-bib-0028] Naud, C.M. , Booth, J.F. , Jeyaratnam, J. , Donner, L.J. , Seman, C.J. , Zhao, M. , Guo, H. and Ming, Y. (2019) Extratropical cyclone clouds in the GFDL climate model: diagnosing biases and the associated causes. Journal of Climate, 32, 6685–6701.

[qj3992-bib-0029] Nuijens, L. and Stevens, B. (2012) The influence of wind speed on shallow marine cumulus convection. Journal of the Atmospheric Sciences, 69, 168–184.

[qj3992-bib-0030] Oertel, A. , Boettcher, M. , Joos, H. , Sprenger, M. , Konow, H. , Hagen, M. and Wernli, H. (2019) Convective activity in an extratropical cyclone and its warm conveyor belt – a case‐study combining observations and a convection‐permitting model simulation. Quarterly Journal of the Royal Meteorological Society, 145, 1406–1426.

[qj3992-bib-0031] Oertel, A. , Boettcher, M. , Joos, H. , Sprenger, M. and Wernli, H. (2020) Potential vorticity structure of embedded convection in a warm conveyor belt and its relevance for large‐scale dynamics. Weather and Climate Dynamics, 1, 127–153.

[qj3992-bib-0032] Oertel, A. , Sprenger, M. , Joos, H. , Boettcher, M. , Konow, H. , Hagen, M. , and Wernli, H . (2021) Observations and simulation of intense convection embedded in a warm conveyor belt – how ambient vertical wind shear determines the dynamical impact. Weather and Climate Dynamics, 2, 89–110.

[qj3992-bib-0033] Saffin, L. , Gray, S. , Methven, J. and Williams, K. (2017) Processes maintaining tropopause sharpness in numerical models. Journal of Geophysical Research: Atmospheres, 122.

[qj3992-bib-0034] Saggiorato, B. , Nuijens, L. , Siebesma, A.P. , de Roode, S. , Sandu, I. and Papritz, L. (2020) The influence of convective momentum transport and vertical wind shear on the evolution of a cold air outbreak. Journal of Advances in Modeling Earth Systems, 12, e2019MS001991. 10.1029/2020MS002183 PMC7757185 33381277

[qj3992-bib-0035] Schäfler, A. , Craig, G. , Wernli, H. , Arbogast, P. , Doyle, J.D. , McTaggart‐Cowan, R. , Methven, J. , Rivière, G. , Ament, F. , Boettcher, M. , Bramberger, M. , Cazenave, Q. , Cotton, R. , Crewell, S. , Delanoë, J. , Dörnbrack, A. , Ehrlich, A. , Ewald, F. , Fix, A. , Grams, C.M. , Gray, S.L. , Grob, H. , Groß, S. , Hagen, M. , Harvey, B. , Hirsch, L. , Jacob, M. , Kölling, T. , Konow, H. , Lemmerz, C. , Lux, O. , Magnusson, L. , Mayer, B. , Mech, M. , Moore, R. , Pelon, J. , Quinting, J. , Rahm, S. , Rapp, M. , Rautenhaus, M. , Reitebuch, O. , Reynolds, C.A. , Sodemann, H. , Spengler, T. , Vaughan, G. , Wendisch, M. , Wirth, M. , Witschas, B. , Wolf, K. and Zinner, T. (2018) The North Atlantic Waveguide and Downstream Impact Experiment. Bulletin of the American Meteorological Society, 99, 1607–1637.

[qj3992-bib-0036] Schemm, S. (2013). *Conveyor belts in idealized moist baroclinic wave life cycles*. PhD Thesis. ETH Research Collection, ETH Zürich, Zürich, Switzerland. 10.3929/ethz-a-7632512

[qj3992-bib-0037] Schmetz, J. , Pili, P. , Tjemkes, S. , Just, D. , Kerkmann, J. , Rota, S. and Ratier, A. (2002) An introduction to meteosat second generation (MSG). Bulletin of the American Meteorological Society, 83(7), 977–992. http://dx.doi.org/10.1175/1520‐0477(2002)083<0977:aitmsg>2.3.co;2.

[qj3992-bib-0038] Selz, T. and Craig, G.C. (2015) Upscale error growth in a high‐resolution simulation of a summertime weather event over Europe. Monthly Weather Review, 143, 813–827.

[qj3992-bib-0039] Shepherd, T.G. (2014) Atmospheric circulation as a source of uncertainty in climate change projections. Nature Geoscience, 7, 703–708.

[qj3992-bib-0040] Spreitzer, E. , Attinger, R. , Boettcher, M. , Forbes, R. , Wernli, H. and Joos, H. (2019) Modification of potential vorticity near the tropopause by nonconservative processes in the ECMWF model. Journal of the Atmospheric Sciences, 76, 1709–1726.

[qj3992-bib-0041] Sprenger, M. and Wernli, H. (2015) The LAGRANTO Lagrangian analysis tool ‐ version 2. Geoscientific Model Development, 8, 2569–2586.

[qj3992-bib-0042] Stevens, B. and Bony, S. (2013) What are climate models missing?. Science, 340, 1053–1054. 23723223 10.1126/science.1237554

[qj3992-bib-0043] Tiedtke, M. (1989) A comprehensive mass flux scheme for cumulus parameterization in large‐scale models. Monthly Weather Review, 117, 1779–1800.

[qj3992-bib-0044] Tung, W. and Yanai, M. (2002) Convective momentum transport observed during the TOGA COARE IOP. Part I: general features. Journal of the Atmospheric Sciences, 59, 1857–1871.

[qj3992-bib-0045] Wernli, H. and Davies, H.C. (1997) A lagrangian‐based analysis of extratropical cyclones. I: the method and some applications. Quarterly Journal of the Royal Meteorological Society, 123, 467–489.

